# Host-Microbiome Synergistic Control on Sphingolipid Metabolism by Mechanotransduction in Model Arthritis

**DOI:** 10.3390/biom9040144

**Published:** 2019-04-09

**Authors:** Xiaoyuan Zhou, Valentina Devescovi, Yuanhua Liu, Jennifer E. Dent, Christine Nardini

**Affiliations:** 1 Department of Neurology, University of California, San Francisco, CA 94158, USA; 2 CAS-MPG Partner Institute for Computational Biology, Shanghai Institutes for Biological Sciences, Shanghai 200031, China; valentina.devescovi@gmail.com (V.D.); yhliu@ips.ac.cn (Y.L.); 3 University of Chinese Academy of Sciences, Beijing 100049, China; 4 Bioinformatics Platform, Institut Pasteur of Shanghai, CAS, Shanghai 200031, China; 5 NORSAS Consultancy Ltd., Hoveton, Norwich, Norfolk, NR128QP, UK; jen.dent@gmail.com; 6 Department of Laboratory Medicine, Division of Clinical Chemistry Karolinska Institute, 17177 Stockholm, Sweden; 7 Scientific and Medical Direction, SOL Group S.r.l, 20900 Monza, Italy; 8 CNR IAC “Mauro Picone”, 00185 Roma, Italy

**Keywords:** rheumatoid arthritis, host-microbiome interaction, sphingolipids metabolism, *Prevotella sp.*, iNKT

## Abstract

Chronic inflammatory autoimmune disorders are systemic diseases with increasing incidence and still lack a cure. More recently, attention has been placed in understanding gastrointestinal (GI) dysbiosis and, although important progress has been made in this area, it is currently unclear to what extent microbiome manipulation can be used in the treatment of autoimmune disorders. Via the use of appropriate models, rheumatoid arthritis (RA), a well-known exemplar of such pathologies, can be exploited to shed light on the currently overlooked effects of existing therapies on the GI microbiome. In this direction, we here explore the crosstalk between the GI microbiome and the host immunity in model arthritis (collagen induced arthritis, CIA). By exploiting *omics* from samples of limited invasiveness (blood and stools), we assess the host-microbiome responses to standard therapy (methotrexate, MTX) combined with mechanical subcutaneous stimulation (MS) and to mechanical stimulation alone. When MS is involved, results reveal the sphingolipid metabolism as the trait d’union among known hallmarks of (model) RA, namely: Imbalance in the S1P-S1PR1 axis, expansion of *Prevotella sp.*, and invariant Natural Killer T (iNKT)-penia, thus offering the base of a rationale to mechanically modulate this pathway as a therapeutic target in RA.

## 1. Introduction

Microbes living in the gastrointestinal (GI) tract have long co-evolved with their hosts, leading to the development of the modern immune and metabolic systems [1]. Mechanisms of interaction between the GI microbiome and the host should, therefore, at a minimum be acknowledged–and ideally, be thoroughly explored–in relation to the understanding and treating of (auto)immune disorders. 

Rheumatoid arthritis (RA), a widely recognized model for autoimmune disorders, has unclear etiology, implicating a combination of genetic [2,3] and environmental factors involved in its onset and progression. Such factors may include bacterial [4,5] and viral infections [6,7], as well as dependency from lifestyle habits, such as exercise and nutrition [8]. Such complexities result in difficulty in controlling the full extent of (adverse) effects of standard therapies for RA, mostly consisting of Disease Modifying Anti-Rheumatic Drugs (DMARDs). Biochemical therapies impact with more or less specificity (biological and conventional DMARDs, respectively) on the host molecular landscape, affecting a broad but yet undetermined number of tightly intertwined pathways and functions, leading to different measures of immunodepression, and with virtually unknown effects on the composition of the GI microbiome [9].

Although it is acknowledged that RA can be influenced by a variety of environmental stimuli (e.g., nutrients and drugs), little is known about the impact of the so-called biologic-free approaches, ranging from fecal microbiota transplantation [10,11] to exploitation of cellular mechano-sensing and mechano-transduction [12]. Recently, however, stimuli varying in nature have been shown as being broadly capable of positively affecting RA: From mechanical stimuli via wound healing in collagen induced arthritis (CIA, [13]) to electrical stimuli in RA via the inflammatory reflex [14]. These non-pharmacological approaches have a tremendous potential to impact on the management of RA patients, yet the exact mechanisms underlying these pioneering efforts need to be further elucidated. 

To shed light on the interplay among mechanosensing (in view of biologic-free approaches), GI microbiome and autoimmunity, we here explore the crosstalk between host and GI microbiome signaling functions in rats affected by model rheumatoid arthritis (CIA) and treated by mechanical stimulation (MS [15,16]). Embracing rather than avoiding the complexity of the disease, we propose a methodology that not only identifies the alterations of synergistic host-microbial functions in this specific case, but also by being general in nature, remains reusable in the investigation of a variety of host-microbiome interactions.

By beginning with a standard differential microbial abundance analysis and moving toward 16S rRNA-inferred functional analysis, we are able to identify significantly altered molecular functions in the microbiome and to search for mirroring/corresponding functional activity in the matching peripheral blood mononuclear cells (PBMC) host samples. This approach leads, overall, to the emergence of results redundant in blood and fecal sampling, pointing to alterations in the sphingolipid metabolism. Results are corroborated by the implementation of a joint analysis of blood transcriptomics and GI metagenomics (computationally overcoming the heterogeneous nature of the data), confirming the importance of the rebalancing effects on the sphingolipids metabolism, synergistically promoted by the host and the GI microbiome. Given the small side of our study, further validation on larger cohort is however necessary.

## 2. Materials and Methods

We here present: (i) Unpublished GI microbiome data, (ii) a novel methodology, and (iii) innovative results that complement and complete the animal study described in our previous paper (batch2, [16]), where the study here presented was solely used as an independent cohort to confirm phenotypic and functional results in blood, GI microbiome data not being available for analysis in our previous study. Accordingly, methods are here briefly recollected (Section 2.1) or newly presented for all the following sections.

### 2.1. Animal Models

**Figure 1:**
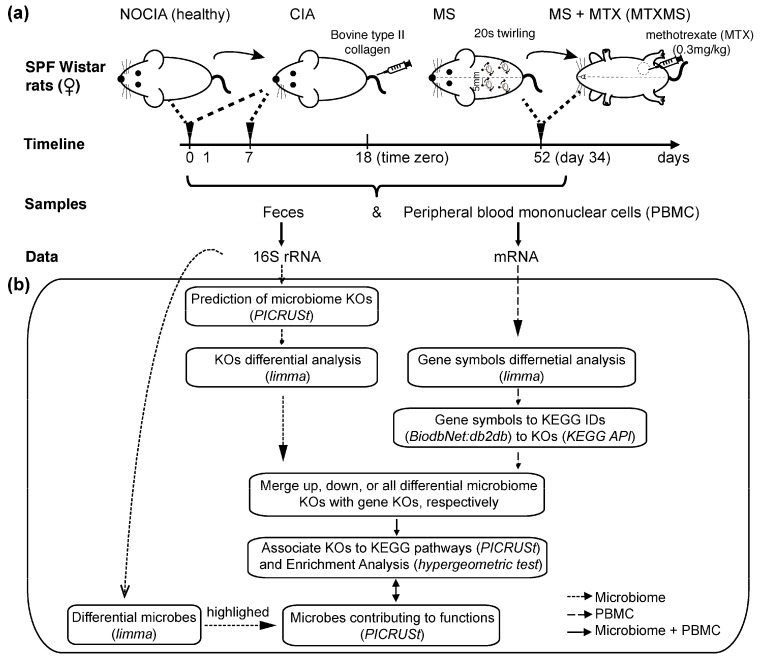
Workflow of the host-microbiome interaction analysis. (**a**) Summary of the experimental design and data collected. See Methods for full details on collagen induced arthritis (CIA) induction; (**b**) Functional integrations of blood genes and gastrointestinal microbiomes. Dashed arrows indicate single *omic* analyses (16S-rRNA microbiome data alone and PBMC transcriptomics alone), and solid arrows represent microbiome and PBMC genes integration steps. NOCIA, non-induced healthy rat; MS, mechanical stimulation; MTX, methotrexate; MTXMS, MTX in conjunction with MS; KEGG, Kyoto Encyclopedia of Genes and Genomes; KO, KEGG orthology; *limma*, Linear Models for Microarray data; PICRUSt, Phylogenetic Investigation of Communities by Reconstruction of Unobserved States.

Graphical representation of the study can be found in Figure 1a. Specific Pathogen Free (SPF) female Wistar rats, purchased from the Animal House Centre of Fudan University (Shanghai, China), were collagen induced to model RA (CIA, [13]) and assigned to two active treatments: Mechanical stimulation (MS, subcutaneous stimulation delivered as acupuncture) and conventional DMARD (methotrexate, MTX, gold standard), in conjunction with the MS therapy (MTXMS). Non-induced rats (NOCIA) represent the healthy reference. Rats were housed five per cage, maintained in an environmentally controlled room with a 12-hour light/dark cycle and given water with standard chow *ad libitum*. The study was approved by the Animal Ethics Committee of Zhongshan Hospital (SYXK 2009-0082), Fudan University (Shanghai, China), performed with methods in accordance with the relevant guidelines and regulations and all measures were taken to minimize animal number and suffering.

Rats underwent anesthesia then immunization by 0.1 mL intradermal injection at the base of the tail, of an emulsion 2 mg/mL Bovine type II collagen (Chondrex) dissolved in 0.05 M acetic acid and mixed 1:1 Complete Freund’s Adjuvant (Sigma-Aldrich, St-Louis, MO, USA). As per protocol to guarantee induction of disease, after seven days, a second immunization was given by a booster injection of Bovine type II collagen prepared as previously described and emulsified 1:1 Incomplete Freund’s Adjuvant (Sigma-Aldrich, DK). Severity of paw inflammation was assessed by (a) a qualitative scoring system (CIA index from 0–4 depending on swelling condition), and (b) quantitative measurements using a thickness gauge (Mitutoyo, Kawasaki, Japan) placed on the hind paw tarsal (dorsal/ventral). Two measurements were collected at each time point to ensure accuracy, with the same frequency of qualitative visual inspection. In accordance with the experimental protocol indications, both the qualitative and the quantitative measurements confirmed that immunization was effective, and all rats developed arthritis, showing the clinical distinctive features of CIA (progressive articular erythema, limbs inflammation, edema) around three weeks post-induction. Onset of the disease was declared at day 18 after immunization for CIA score ≥ 2 and treatments were initiated. Rats were examined for visual signs of disease every other day, starting from day eight (18 time-points in total), macroscopic evidence of increase in hind paw size was noted and paw score determined and reported in Data S1.

The MS consists of a mechanical stimulation by insertion and 20 s clockwise twirling (at the beginning of each treatment, 20 min long) of a ring-headed thumbtack-like stainless-steel needle (ϕ0.25 mm × 2 mm, diameter × length, Hwato, Suzhou, China). Mechanical stimulation was applied bilaterally every day on points located at each side of the lower back (between the second and the third lumbar vertebra) and between the tibia and fibula (at approximately 5 mm lateral and 5 mm lower to the anterior tubercle of the tibia [17]). These points are also known in traditional Chinese medicine as “Shenshu” (BL 23) and “Zusanli” (ST 36), respectively. The MTXMS group received both therapies, i.e., MS daily and MTX by intraperitoneal injections of 0.3 mg/Kg of drug in sterile saline once a week. 

Whole blood sampling for high-throughput screens was carried out before therapy for the CIA and NOCIA groups and at 34 days for the MS and MTXMS groups. Total RNA extraction and pre-processed transcriptomic mRNA profiles were analyzed as described in Reference [16].

### 2.2. Fecal Sample Collection

Fecal specimens were collected in sterile vials, rapidly frozen at −20 °C, and further stored at −80 °C. Microbial DNA was extracted from fecal samples using the E.Z.N.A.® Soil DNA Kit (Omega Bio-tek, Norcross, GA, U.S.) according to the manufacturer’s protocols. The V1-V3 region of the bacteria 16S ribosomal RNA gene was amplified using broadly conserved primers 27F 5’-(CGTATCGCCTCCCTCGCGCCATCAG-3’ 5’-AGAGTTTGATCCTGGCTCAG)-3’ and 533R 5’- (CTATGCGCCTTGCCAGCCCGCTCAG- 3’ -MID tags-5’-ATTACCGCGGCTGCTGGCA)-3’. Polymerase chain reactions of this region were performed in a 20 μL mixture containing 4 μL of 5 × FastPfu Buffer, 2 μL of 2.5 mM dNTPs, 0.8 μL of each primer (5 μM), 0.4 μL of FastPfu Polymerase, and 10 ng of template DNA. The amplification program consisted of an initial denaturation step at 95 °C for 2 min, followed by 25 cycles, where one cycle consisted of 95 °C for 30 s (denaturation), 55 °C for 30 s (annealing) and 72 °C for 30 s (extension), and a final extension of 72 °C for 5 min.

### 2.3. Microbial 16S rRNA Sequencing

After purification using the AxyPrep DNA Gel Extraction Kit (Axygen Biosciences, Union City, CA, U.S.) and quantification using QuantiFluor™ -ST (Promega, Madison, WI, U.S.), a mixture of amplicons was used for pyrosequencing on a Roche 454 GS FLX+ Titanium platform (Roche 454 Life Sciences, Branford, CT, U.S.) at a depth of 15,000 reads per sample according to standard protocols at Majorbio Bio-Pharm Technology Co., Ltd., Shanghai, China. Raw 16S rRNA gene sequences were analyzed for sequences quality control, sequences selection based on samples’ barcode identifiers and sequences alignment (SILVA database, SSU106 [18]) to trim chimeras. Operational Taxonomy Units (OTUs) were classified by mapping trimmed 16S rRNA sequences against Greengenes reference OTUs (representative 16S RNAs) [19] using *blast* at 97% identity with QIIME-1.7.0 [20] and assigned a taxonomy [21], in the form of a table having OTUs as rows, samples as columns and OTUs abundances in each cell.

### 2.4. General Method

Figure 1b summarizes the workflow of our proposed approach to integrate host (blood mRNA) and gut microbiome (16S rRNA) data. Every step is detailed in the subsections below. 

#### 2.4.1. OTUs Expansion in KEGG Orthology

This step allows for the inference of metagenomic information from 16S rRNA-seq data. PICRUSt (Phylogenetic Investigation of Communities by Reconstruction of Unobserved States [22]) version 1.0.0 was applied to normalize the OTU table produced in Section 2.3 by known/predicted 16S-rRNA gene copy numbers and to predict metagenome abundances and functions using KEGG (Kyoto Encyclopedia of Genes and Genomes) orthologies (KOs, [23]) based on pre-computed gene content information in PICRUSt. The predicted KEGG orthologies referred to, from this point on, as GI KOs, were further collapsed into KEGG pathways within PICRUSt for functional analysis.

#### 2.4.2. Statistical Analysis

Pairwise comparisons within treatments and between time points were performed on: (i) Gastrointestinal microbiome OTUs for identification of genera abundance variations, and (ii) Gastrointestinal KEGG Orthologies for microbiome functional analysis; (iii) Peripheral blood mononuclear cell transcripts (mRNAs) for further gene symbol conversion and host functional analysis. To limit bias and guarantee clarity of the process the same functional analysis pipeline was uniformly applied to all 3 cases (OTUs, GI KOs, PBMC transcripts): Linear Models for Microarray Data (*limma* [24]) and Voom conversion for microbial data (i,ii) [25] and *limma* for PBMC transcripts (iii). To identify meaningful features a combination of uncorrected *p* value (*p* < 0.05) and fold change (FC ≥ 2) was used and log-FC confidence intervals [26] were considered for selected differential features (Appendix A). This approach allows us to grant that potentially meaningful features are retained for further investigation, despite the limited sample size of our experiment.

#### 2.4.3. Gene Symbols Conversion into KEGG Orthologies

To allow comparison between Genes Symbols from PBMC mRNAs and GI KOs, PBMC mRNAs were also converted from “Gene Symbol” to “KEGG gene ID” with a Rattus taxon ID 10116 by using the database-to-database conversion tool bioDBnet:db2db [27]. KEGG gene IDs were then linked to KOs and pathways through the KEGG API and are referred to, from this point on, as PBMC KOs.

#### 2.4.4. Functional Analysis

Differential GI KOs, PBMC KOs as well as their union were analyzed by a modified Fisher’s exact test to calculate EASE score (*p* value) for the identification of enriched KEGG pathways [28,29]. OTUs contributing to the KOs enriched in specific KEGG pathways were identified within PICRUSt.

### 2.5. Data and Materials Availibility

16S rRNA raw data are available in the National Center for Biotechnology Information Sequence Read Archive database (Accession Number: SRP034535). PBMC mRNA data are available at the National Center for Biotechnology Information Gene Expression Omnibus (GSE58456).

## 3. Results

**Figure 2:**
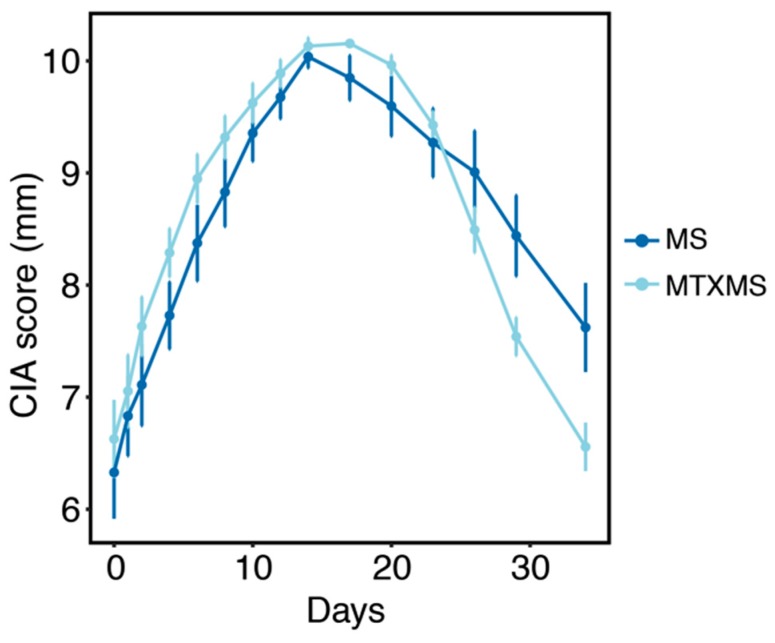
Phenotypic characterization. Standard clinical parameters (paws’ thickness, CIA score, mm) in the mechanical stimulation (MS) and MTX in conjunction with MS arms (MTXMS) over time (34 days) drawn from published data (Data S1), excerpt from [16]. Data are presented as mean ± SEM (Standard Error of Mean).

As for section Material and Methods the founding result of the study, i.e., effectiveness of the proposed therapies from a phenotypic standpoint are recalled from our previous work [16] in Figure 2, all following innovative analyses’ results, investigating the biochemical activity behind such phenotypes are detailed from Section 3.2.

### 3.1. Collagen Induced Arthritis Phenotype

Prior to performing any molecular analyses, the effectiveness of the therapeutic approaches in standard phenotypic terms is reported (Figure 2 and Data S1). The study (Figure 1) was designed with particular attention to the translational aspects of the research, mimicking clinical routine (see clinical trial NCT01619176 at www.clinicaltrial.gov) to assess the effects of MTX and the addition of the subcutaneous mechanical stimulation (MTXMS). This expands our earlier results on the general deflation of the inflammatory landscape and the activation of molecular wound healing/homeostatic functions assessed in MS alone, by comparing with MTX alone, and control groups of placebo for MTX (sterile saline solution) and control inhalational anesthetic (ANE, isoflurane, versus ether used in PLA, MS and MTX during animals’ blood sampling) [16].

### 3.2. Microbial Taxa Differential Analysis

**Figure 3:**
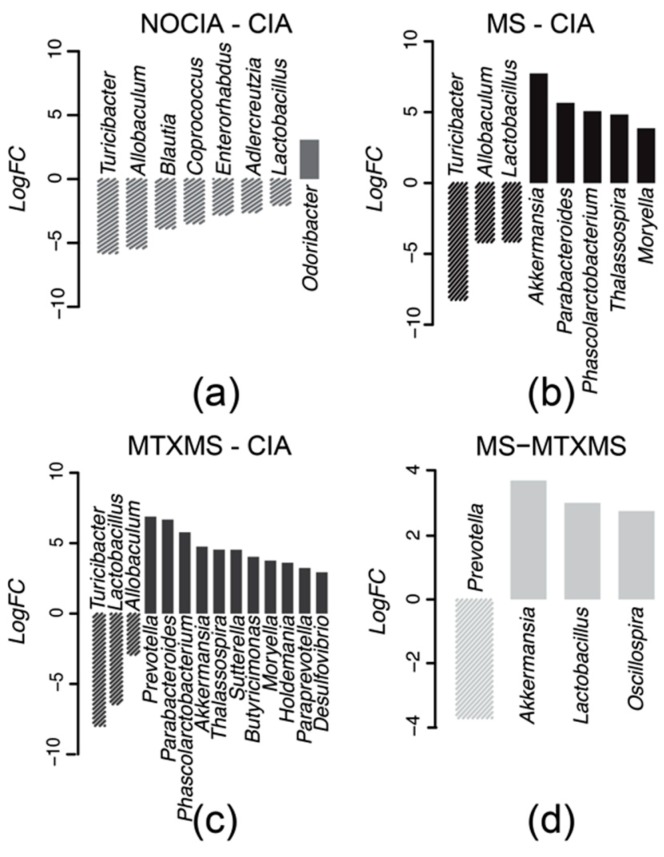
Gastrointestinal microbial differential analysis. Therapy-wise genera differential analyses between (**a**) NOCIA and CIA; (**b**) MS and CIA; (**c**) MTXMS and CIA and (**d**) MS and MTXMS. Data are represented by log fold change (log-FC) with *limma* (FC ≥ 2, *p* ≤ 0.05).

**Figure A1:**
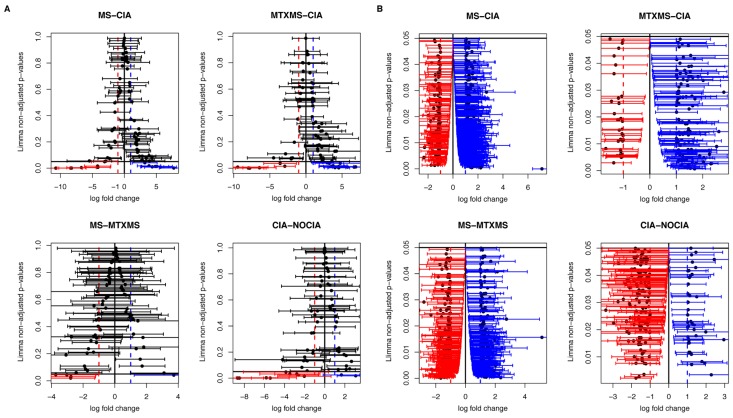
Log fold change confidence interval calculated for (**A**) all gut microbiome genera and (**B**) differential genes (*p* < 0.05 and FC ≥ 2) for a better visualization. Increased (log-FC ≥ 1) and decreased (log-FC ≤ −1) features were labeled in blue and red, respectively.

Results of the differential analysis along with the log fold change confidence interval estimation (Appendix A and Figure A1), which allows quantification of the variations between healthy (NOCIA) and CIA induced rats’ GI microbiomes (Figure 3a), as well as variations upon the delivery of the MS and MTXMS therapies (Figure 3b,c, Data S2) highlighted three genera of interest: *Turicibacter*, *Allobaculum* and *Lactobacillus*. Initially, all three were present with a significant increase in the CIA arm in comparison to the healthy baseline (Figure 3a). Subsequently, these same genera appear to be significantly reduced by both the MS and MTXMS treatments (Figure 3b,c), thus representing a common effect of the two therapies, coherent with previous results [16].

The differential effects of the two active therapies (MS vs MTXMS, Figure 3d) let emerge *Prevotella* as the only markedly and significantly more abundant genus in the MTXMS vs MS comparison. This is coherent with our previous findings [16] (Appendix A and Table S1) which show how, upon treatment of CIA rats, a significant expansion of *Prevotella* is visible only in the MTX arm and not in the MS arm. Species from the *Prevotella* genus (*P. copri*) are well recognized actors in the onset of arthritis [30], although this specific strain is not present in our data.

### 3.3. Single Omic Functional Analysis

#### 3.3.1. Microbiome Side, Gastrointestinal KEGG Orthologies

**Figure 4:**
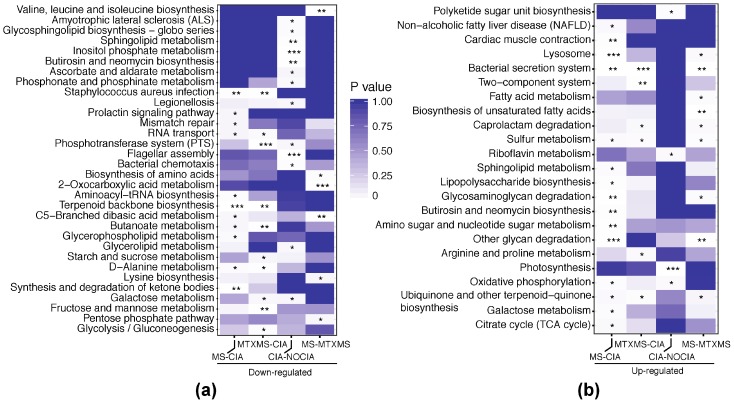
KEGG functional analysis of microbiome. In the rows the enriched functions and in the columns the conditions compared, labeled by increased (Up) or reduced (Down) abundance in each comparison. (**a**) KEGG pathways enriched by down-regulated microbiome KOs; (**b**) KEGG pathways enriched by upregulated microbiome KOs. Data are represented by modified Fisher’s exact *p* values, *p* ≤ 0.001 with ***, *p* ≤ 0.01 with **, *p* ≤ 0.05 with *.

Table 1 (excerpt from Data S3 with complete results) highlights in the first two columns the statistically significant results characterized by opposite behaviors (defined as altered biological functions, inferred from 16S-rRNA sequencing data, see Materials and Methods) in CIA and MS. In particular we observe a lack of activity referring to *Galactose metabolism*, *Sphingolipid metabolism* and *Butirosin and neomycin biosynthesis* (i.e., a reduction of genes -KOs- enriched in these functions, see Materials and Methods) in CIA versus the healthy baseline (Figure 4a), all of which were then significantly restored upon MS treatment (Figure 4b). *Butirosin and neomycin* are bacterial endogenous aminoglycoside antibiotics, the emergence of which, in our analysis, is likely owing to their glycoside (sugar-derived) molecule portion. It is noted that the galactose metabolism is necessary for the biosynthesis of glycosphingolipids that are secondary/intermediate products of the sphingolipids cascade, hence closely tied to the aforementioned sphingolipid metabolism.

Further, the sphingolipid metabolism, unique to the MS vs. CIA arm, is backed by the Glycosaminoglycan degradation emerging in the MS versus MTXMS comparison (Data S3). Glycosaminoglycan content was seen to increase in blood and arthritic tissues during the progression of RA, as reported by several studies, suggesting a causal relationship with the disease [31,32]. This indicates that MS may also correlate with the control of this disrupted pathway.

Finally, for the MTXMS arm we observe the involvement of carbohydrate metabolism, particularly in starch and sucrose metabolisms, Glycolysis/Gluconeogenesis, as well as fructose and mannose metabolisms. This is in line [33] with the effects of MTX, known to be coupled with a change in tissue-specific metabolism and energetic consumption, supplied by the catabolism of simple and complex carbohydrates (glycolysis of mono-, and poly-saccharides) or their de novo synthesis from amino acids (gluconeogenesis).

#### 3.3.2. Host Side, Peripheral Blood Mononuclear Cell KEGG Orthologies

**Figure 5:**
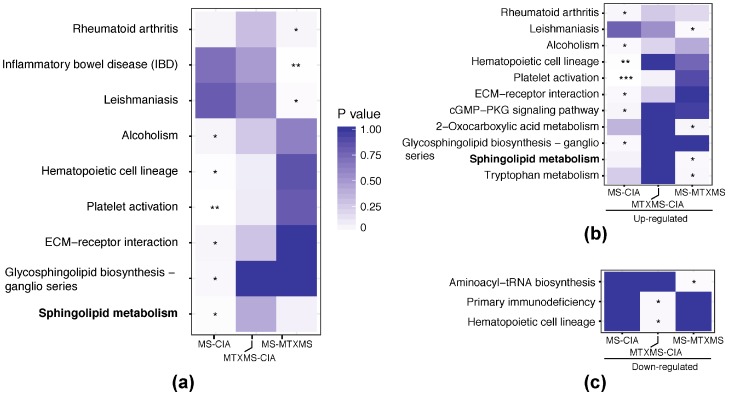
KEGG functional analysis of peripheral blood mononuclear cell genes. In the rows the enriched functions and in the columns the conditions compared, labeled by all differential, or increased (Up) or reduced (Down) abundance in each comparison. (**a**) KEGG pathways enriched by all differential PBMC genes; (**b**) KEGG pathways enriched by up-regulated differential PBMC genes; (**c**) KEGG pathways enriched by down-regulated differential PBMC genes. Data are represented by modified Fisher’s exact *p* values, *p* ≤ 0.001 with ***, *p* ≤ 0.01 with **, *p* ≤ 0.05 with *.

The independent functional analysis run on PBMC KOs revealed mirroring findings to the GI KOs as can be observed in Figure 5 and Data S4 (GO analysis from our previous and current independent experiments), where we read that in MS only (in comparison to both CIA and MTXMS) the sphingolipid metabolism is also, and distinctively, present. 

This is coherent with the emergence of *Cardiac muscle contraction* specific to MS (Data S3), owing to the involvement of the sphingosine-1-phosphate (S1P) molecular pathway in regulating cardiac cell differentiation and contractile functions of coronary arteries and heart rate [34], which fostered application of sphingosine-1-phosphate receptor 1 (S1PR1)-modulating drugs (FTY720) to vascular chronic inflammatory diseases [35].

### 3.4. Host-Microbiome Interaction, KEGG Orthology Joint Analysis

In this section, we explore the results of the KOs produced in both districts (blood and gut-intestine) at the same time points and under the same conditions for identification of synergistically enriched differential functions. With this approach, it is possible to deconvolute from the joint KO significantly enriched terms the PBMC transcripts and the GI OTUs that contribute to the statistical significance of the KO term.

In this joint analysis (Table 2 and Data S5), again, alteration of the sphingolipid metabolism emerges as a distinctive feature of MS. This feature appears both in the comparison versus CIA and versus MTXMS. Compared with CIA, it is associated with the overexpression of host PBMC genes *Acer2*, *Asah1*, and *B4galt6*, as well as with expansion of *Akkermansia*, *Parabacteroides*, *Moryella*, and the reduction of *Lactobacillus*, *Turicibacter*, *Allobaculum*, the latter being an already mentioned common effect of both the MS and MTXMS therapies. Versus MTXMS it is attributed to the increased presence of *B4galt6*, *Ppap2c* and *Sptlc1* transcripts, to expansion of *Akkermansia*, *Lactobacillus*, *Oscillospira*, and reduction of *Prevotella*, in the MS arm only.

## 4. Discussion

Our results identify several promising areas in terms of mechanistic understanding of the phenomena elicited by biomechanical stimuli in a chronic inflammatory environment, and of their therapeutic potential. We discuss these findings here in more detail and highlight that these results deserve to be further investigated with larger studies, ours being of limited size.

### 4.1. Collagen Induced Arthritis Dysbiosis

Despite CIA modeling the altered host immune response typical of RA with no specific attention to the underlying dysbiosis, inflammatory triggers are well-known to alter the GI microbiome composition [36], and therefore considerations on the impact of CIA on the microbiome remain of value. Specifically, the three genera highlighted by the differential analysis of the CIA model have already been found in association with RA: *Turicibacter* is reported to be expanded in RA patients [5], *Allobaculum* to be highly abundant in arthritis-susceptible HLA transgenic mice compared to arthritis-resistant mice [37] and *Lactobacillus* to be over represented in RA [38,39,40], confirming the importance of the inflammatory factor in this disorder. This offers a first taxonomy of the effects of CIA on the GI microbiome, a fact that will have to be studied with more care, including a larger sample size, to offer a model of the disease that is able to mimic the growing knowledge of the disorder and offers research results that are guaranteed to be translatable.

### 4.2. The Sphingolipid Metabolism—Etiopathogenesis of Rheumatoid Arthritis

Both single *omics*’ (GI microbiome KOs and PBMC transcriptome, separately) functional analyses and the host-microbiome joint analysis revealed concordant variations in the sphingolipid metabolism. The bioactive metabolites of this pathway (in particular ceramide, sphingosine and the ’pleiotropic’ factor S1P, mutually converted in a dynamic interchange) have important roles in stimulus/agonist-mediated signaling, which regulate diverse and even opposite cellular functions, ranging from cell proliferation to apoptosis, angiogenesis and cell migration to control of immunity and inflammation. Importantly, fluctuations in the rate of extracellular S1P concentrations (and, consequently, S1P cell receptors’ upregulation or downregulation) are crucial cues in controlling the inflammatory and immune responses, with relevance in a number of diseases including cancer and autoimmune and inflammatory disorders [41]. Relevant to the problem under investigation, the axis consisting of S1P and its receptor (S1P-S1PR1) emerged as a central regulator of T and B lymphocytes egress from lymphoid tissues in response to the S1P gradient, controlled via the activation of its receptor S1PR1. Namely, effector lymphocytes upregulate S1PR1 to be responsive to the S1P gradient and either migrate along to inflamed sites or reduce the receptor expression when residing in a specific compartment [42]. Specifically, the S1P-S1PR1 system has been demonstrated to control not only T cells (CD4+ Th) migration and tissue distribution, but also initiation of early events of differentiation into effector states (e.g., proliferation and secretion of interferon gamma (IFN-γ) and interleukin-4 (IL-4). This has implications in diseases with impaired lymphocytes’ immune-mediated responses, as it is the case in inflammatory chronic autoimmunity [43] like RA. Moreover, the S1P-S1PR1 axis was also proven to regulate migratory behaviors of bone-resorbing cells (osteoclasts), critically controlling their differentiation and dynamically modulating bone mineral homeostasis, with the role of S1PR1 functional antagonist becoming relevant also in bone destructive disorders like RA [44].

### 4.3. The Sphingolipid Metabolism—Potential Therapeutic Target of Mechnical Stimulation

The power of the joint analysis allows one to identify directly, with no a priori (unsupervised approach), both the host transcripts and the GI bacteria that jointly contribute to the alteration of the sphingolipid metabolism by MS. Acer2 and Asah1, two genes hydrolyzing the sphingolipid ceramide into sphingosine and free fatty acid, were increased in MS compared to CIA. Specifically, exclusive results (i.e., not shared by other comparisons) between the two treatments MS-MTXMS are discussed in this section. 

Before entering into the details of our findings, we recall in this paragraph what is known in literature. Regarding the host components, the three emerging transcripts (Ppap2c, Sptlc1, B4galt6) cover crucial functions in various aspects of the whole pathway: Ppap2c hydrolyzes ceramide-1-phosphate (C1P) and S1P, which are among the major bioactive metabolites of this pathway and Sptlc1 is the Serine Palmitoyltransferase (SPT) Long Chain Base Subunit 1, with SPT being the enzyme at the origin of the sphingolipid metabolism. With respect to the interaction with microbial components, the third transcript, B4galt6, is involved downstream of the sphingolipid pathway and shares an additional and important role in response to pathogens, shown to affect the activity of invariant Natural Killer T (iNKT) cells [45]. Growing evidence converges towards the importance of this crosstalk between pathogens and iNKT activity. In particular, Bacteroides, Prevotella, and Porphyromona are known to be equipped with the ability to metabolize sphingolipids [46], with Bacteroides being able to negatively regulate iNKT [47] and *Prevotella* being distinctively increased in iNKT deprived animals affected by chemically induced colitis [48]. 

Having recollected these findings, we can speculate on the relevance of the sphingolipid metabolism emerging in our results as a gluing theme to all the above-mentioned fact, hypothesizing that the expansion of *Prevotella* in new onset RA [30], tied in our results to alterations of the sphingolipid metabolism, is also possibly responsible for the excessive negative regulation of iNKTs, leading to the iNKT-penia typical of RA patients [49]. This is also in line with *Porphyromona gingivalis* being another well-known initiator of RA and a sphingolipid equipped bacterium, clarifying the well-known pathogenetic properties of *P. gingivalis* towards RA, in addition to the known citrullination issue [50]. 

Our results therefore suggest that these three known hallmarks of RA/CIA (imbalanced S1P-S1PR1 axis, iNKT-penia, expansion of *Prevotella*) are different aspects of a same phenomenon. A phenomenon that remains uncontrolled by the MTX associated treatment (MTXMS) marked by *Prevotella* expansion, opposite to what happens in the MS arm. In fact, the control on inflammation associated with the wound healing response–that is, a consequence of the mild MS mechanical injury [16]–can indirectly promote the growth or reduction of specific strains of bacteria: persistence or control of the inflammatory condition enhances or reduces the availability of substrates that only opportunistic bacteria can exploit, provoking their expansion in spite of other more eubiotic genera [1]. The global host-microbiome response will depend on the bacteria’s ability to process the excess of cytokine products and reactive species, and, from there, these species’ abundance readjustment can alter the ability to produce the sphingolipids needed to properly control iNKT activation [47].

Overall, the method presented has enabled the identification of the therapeutic potential of MS on model RA by regulating the sphingolipid metabolic pathway, a pathway that is active in both host PBMC and gut-intestinal microbes. The approach has enabled us to put in an integrated interpretative frame, for the first time, four important and, up to now, relatively unrelated findings: (i) The sphingolipids metabolism, biased in the RA/CIA host towards a dysregulated inflammatory response [42]; (ii) the expansion of *Prevotella sp.* in new onset RA patients [30]; (iii) the known iNKT-penia typical of RA patients [49]; and (iv) the ability of symbionts to maintain homeostasis by subtle control of the iNKT cells activation via sphingolipids metabolism [47].

The exact chain of cause and effects cannot be inferred from the evidence offered by our experiment and, to date, very minimal literature exists on the topic, including recent findings on the role of the sphingolipid metabolism in the regulation of integrins mechanosensing [51]. Independently integrating these pioneer findings, our approach can suggest likely actors/actuators of the alterations observed, offering better defined hypotheses to design subsequent and more specific experiments to use this pathway as RA therapeutic target, highly needed in the current clinical and basic research, with the potential to offer unprecedented improvement in the management of chronically inflamed patients.

## Figures and Tables

**Table 1:** KEGG pathways enriched by more and less abundant microbiome KEGG orthologies.

KEGG Pathway	CIA vs. NOCIA		MS vs. CIA		MTXMS vs. CIA	
	Up	Down	Up	Down	Up	Down
Galactose metabolism	0.7426	***0.0437***	***0.0194***	0.4272	0.3884	***0.0357***
Sphingolipid metabolism	1	***0.0098***	***0.0209***	1	0.6777	1
Butirosin and neomycin biosynthesis	1	***0.0067***	***0.0097***	1	0.1246	1
Enrichment by *p* values (*p* ≤ 0.05 in bold)						

**Table 2:** Sphingolipid metabolism pathway enriched by PBMC transcripts and microbiome KOs, excerpt from Data S5.

KEGG Pathway	Gene	Microbe	Comparison
Sphingolipid metabolism	***Acer2***, ***Asah1***, ***B4galt6***, *Sptlc1*	***Akkermansia***, ***Parabacteroides***, *Lactobacillus*, *Turicibacter*, *Allobaculum*, ***Moryella***	MS-CIA_ALL
	***Acer2**, **Asah1**, **B4galt6***	***Akkermansia***, ***Parabacteroides***, *Lactobacillus*, *Turicibacter*, *Allobaculum*, ***Moryella***	MS-CIA_UP
	***B4galt6***, ***Ppap2c***, ***Sptlc1***	***Akkermansia***, *Prevotella*, ***Lactobacillus***, ***Oscillospira ***	MS-MTXMS_ALL; MS-MTXMS_UP
	***Acer2***	*Blautia*, *Lactobacillus*, *Turicibacter*, *Coprococcus*, ***Odoribacter***	NOCIA-CIA_ALL; NOCIA-CIA_UP

Terms in bold indicate an increase, and regular indicate a decrease. Microbes are ordered by their microbiome contribution to the KEGG pathway from high to low. Notations _ALL, _UP, _DOWN refer respectively to the usage of all KOs, incremented only KOs, reduced only KOs.

## Supplementary Materials

The following are available online at https://www.mdpi.com/2218-273X/9/4/144/s1. Data S1: Categorial and continuous phenotypic data for the progression of CIA, Data S2: Differential fecal 16SrRNA data analysis, Data S3: KEGG pathways significantly enriched by differential microbiome KEGG orthologies, Data S4: Differential PBMC mRNA analysis and functional enrichment, Data S5: Significantly enriched KEGG pathways by both PICRUST predicted metagenome KOs and blood gene, Data S6: Microbial differential analysis in previous data [16].

## Appendix A

Log fold changes (log-FCs) of differential microbiome genera and PBMC genes between two biological conditions were calculated in *limma* (Materials and Methods). To evaluate the biological relevance of differential features in terms of a fold change larger than 2, we applied the unadjusted log-FC confidence intervals, as described in [26], to classify the features in the category of “Log fold change statistically significant and (probably/not) biologically relevant” based on the location of their confidence intervals relative to a log-FC threshold, which in our case is 1 (Figure A1). The upper log-FC confidence limits selected differential features exceed the threshold, which indicates a log fold change statistically significant with potential biological relevance.

The microbial structure analyses in previous independent data [16] confirmed that Lactobacillus was more abundant in the CIA than NOCIA arm, while it was significantly reduced by MTX, as it happens now in the MTXMS arm (Table A1, Data S6). The MS therapy correlates with an increase of *Lactobacillus* compared to either MTX or MTXMS. As it has been reported, some species in the *Lactobacillus* genus, such as *L. casei*, *L. plantarum* and *L. rhamnosus* are (opportunistic) pathogens [52,53]. This, however, is not observed in our data. Other species, like *L. delbrueckii*, are probiotics, leading to the dual (eubiotic or pathogenic) functions of *Lactobacillus* [54]. This should be further explored in more details on species classification. *Prevotella*, a recognized player in the onset of rheumatoid arthritis, was expanded in the MTX arm compared to CIA in our previous experiment [16], supporting this same trend in MTXMS versus MS (Table A1).

**Table A1:** Differential genera in GI-PBMC integration in our previous experiment (batch1, [16]) and in the current batch (batch2), highlighted from Data S6.

Genus	Batch_Current				Batch_Previous			
	MS/CIA	MTXMS/CIA	CIA/NOCIA	MS/MTXMS	MS/CIA	MTX/CIA	CIA/NOCIA	MS/MTX
*Lactobacillus*	−4.137 *	−6.446	2.03	3.028	-	−2.021	2.708	2.504
*Prevotella*	-	6.887	-	−3.725	-	2.108	-	-
*Allobaculum*	−4.18	−2.929	5.418	-	2.757	2.429	-	-
*Odoribacter*	-	-	−3.078	-	-	-	2.917	-
*Blautia*	-	-	3.862	-	4.695	3.369	−3.453	-
*Phascolarctobacterium*	5.068	5.768	-	-	-	3.684	-	-
*Adlercreutzia*	-	-	2.6	-	−2.002	-	-	-

* Numbers shown in the table are log-converted fold changes of the differential (34 days versus before therapy) normalized genera abundances.
